# Rapid recurrence and bilateral lungs, multiple bone metastasis of malignant solitary fibrous tumor of the right occipital lobe: report of a case and review

**DOI:** 10.1186/s13000-015-0318-9

**Published:** 2015-07-09

**Authors:** Zhengrong Wu, Hongjun Yang, Desheng Weng, Yanqing Ding

**Affiliations:** Department of Pathology, School of Basic Medical Sciences; Department of Pathology, Nan Fang Hospital, Southern Medical University, Guangzhou, 510515 China; State Key Laboratory of Oncology in South China; Collaborative Innovation Center for Cancer Medicine, Sun Yat-sen University Cancer Center, Guangzhou, 510060 China

**Keywords:** Intracranial tumor, Malignant solitary fibrous tumor, Clinicopathology, Diagnosis, Differential diagnosis

## Abstract

**Background:**

Intracranial malignant solitary fibrous tumor (MSFT) is extremely rare. The authors report a case of MSFT of the right occipital lobe with a rapid recurrence and bilateral lung, multiple bone metastasis.

**Case presentation:**

The patient was a 25-year-old male presenting with headache, nausea and visual disturbances without obvious cause. Three times right-side occipital craniotomies were performed and two times postoperative conformal radiotherapy were administered within one year. 4 months after the third time of right-side occipital craniotomy, the patient felt right chest pain and neck pain. Positron emission tomography/computed tomography (PET/CT) showed tumor recurrence of the right occipital lobe and bilateral lung metastasis, multiple bone metastasis including: vertebrae, libs, the left iliac wing, sacrum, the right ischium and upper parts of both femurs. Ultrasound guided puncture biopsy of left-side back of the neck and CT guided puncture biopsy of the third lumbar vertebra were performed. General sample showed grayish white or grayish red with irregular shape. Histopathologically, the tumor was composed of areas of alternating hypercellularity and hypocellularity with spindle-shaped cells, which arranged as fascicular, storiform pattern or patternless pattern, with intervening irregular eosinophilic collagen bundles. Some areas showed hemangiopericytoma-like perivascular pattern and perivascular hyalinization. Tumor cells were pleomorphic with mitotic counts of more than 4 per 10 high power fields and showed coagulative necrosis. Immunohistochemically, tumor cells were diffusely positive for vimentin and CD99, focal positive for CD34, bcl-2 and Actin. Ki-67 labelling index was more than 40 %. The final pathological diagnosis was MSFT of the right occipital lobe, metastatic MSFT of left-side back of the neck and the third lumbar vertebra.

**Conclusion:**

The MSFT of the right occipital lobe with recurrence and bilateral lung, multiple bone metastasis is extremely rare. Although intracranial MSFT is extremely rare, it should be considered in the differential diagnosis. Definite diagnosis depended mainly on pathological morphology and immunohistochemistry. The prognosis of MSFT is poor due to recurrence and metastasis. Complete resection of intracranial MSFT is difficult, and carful follow-up is needed.

## Background

Solitary fibrous tumor (SFT) is a rare spindle cell tumor of mesenchymal origin and was first differentiated from mesothelioma in 1931 as primary neoplasms of the pleura [1]. Most SFT occur in the pleural cavity, however, it can be found throughout the body. The exact incidence in the central nervous system (CNS) is not clear, but is reported to be low [2]. Meanwhile, the diagnosis of intracranial SFT can be difficult because of its rarity or histological and radiological resemblance to other more common brain tumors, such as meningioma or hemangiopericytoma. Unlike the clinical behavior of thoracic SFT, of which 37 % were malignant, it has been well known that the vast majority (more than 94 %) of SFT involving CNS are benign and seldom recur or metastasize [3]. Since Ng HK et al. first reported a case of metastatic SFT of the meninges in 2000, 10 additional definite cases have been published in the literature (see Table 1) [4–14]. Report on a malignant SFT (MSFT) of the right occipital lobe is extremely rare. Here we report a very rare case of MSFT of the right occipital lobe with a rapid recurrence and metastasis.

**Table 1:** Clinicopathological features of intracranial malignant solitary fibrous tumors

Report	Age/Sex	Location	Histology	Follow-up
Ng et al. 2000 [4]	55/F	posterior fossa/cerebellum	cellular pleomorphism, no mitoses in PT; 3–4 mitoses/10HPFs in RT, the same Ki-67 LI of 3 %	3 recurrences over 10years, metastasis to the soft tissues and lungs
Barron et al. 2001 [5]	61/F	frontal	hypercellularity, marked pleomorphism, 4 mitoses/10 HPFs	10 months (postoperative radiation herapy given)
Martin et al. 2001 [6]	71/F	dura around the sagittal sinus	marked pleomorphism, 6 mitoses /10 HPFs	death 1 month after the diagnosis
Kim et al. 2004 [7]	55/M	posterior fossa	hypercellularity and more than 4 mitoses/10HPFs, pleomorphism, hemorrhage, and necrosis	9 years,disseminated to the spine, lung, and liver
Miyashita et al. 2004 [8]	63/F	falcotentorial	the MIB-1 LI increased from less than 1 % in the PT to 13 % in the latest tumor	several recurrences over 15 years, dissemination to the cerebrospinal fluid
Ogawa et al. 2004 [9]	70/F	meninge	Ki67 LI and mitosis rates were 3.1 ± 1.2 % and less than 1/10HPF in the PT and 16.1 ± 6.4 % and 6/10HPF in the last (5th) one	4 recurrences over 26 years, metastasis to the right lung and focal invasion into the cerebellum
Pizem et al. 2004 [10]	60/F	dura-based, left frontal	the mitoses increased from less than 1/10HPF in the PT to 16/10HPF in the latest tumor	4 recurrences over a 30-year period
Lawlor et al. 2008 [11]	77/M	dural-based, left parietal	invasion into the sagittal sinus, 19 mitoses/10HPFs, marked amianthoid fibre deposition	not known
Hu et al. 2009 [12]	54/M	dural-based, left occipital	hypercellularity, rare mitoses in PT, >20 mitoses/10 HPFs in RT	died 16 month later due to recurrence and metastasis to bilateral lungs
Zhang et al. 2010 [13]	49/F	pineal region	hypercellularity, 5 mitoses/10HPFs, and MIB-1 LI (7.3 ± 1.8 %)	10 months (postoperative radiotherapy not performed)
Choi et al. 2011 [14]	39/F	left occipital lobe	hypercellularity, pleomorphism, Ki-67 LI increased from 10 % (average, 1 %) in PT to 25 % in RT with infiltrative brain invasion and 15 mitoses/10HPFs	recurrences over a 6-months period

*M* male, *F* female, *HPF*, high-power field, *LI*, labeling index, *PT*, primary tumor, *RT*, recurrent tumor

## Case presentation

**Fig. 1:**
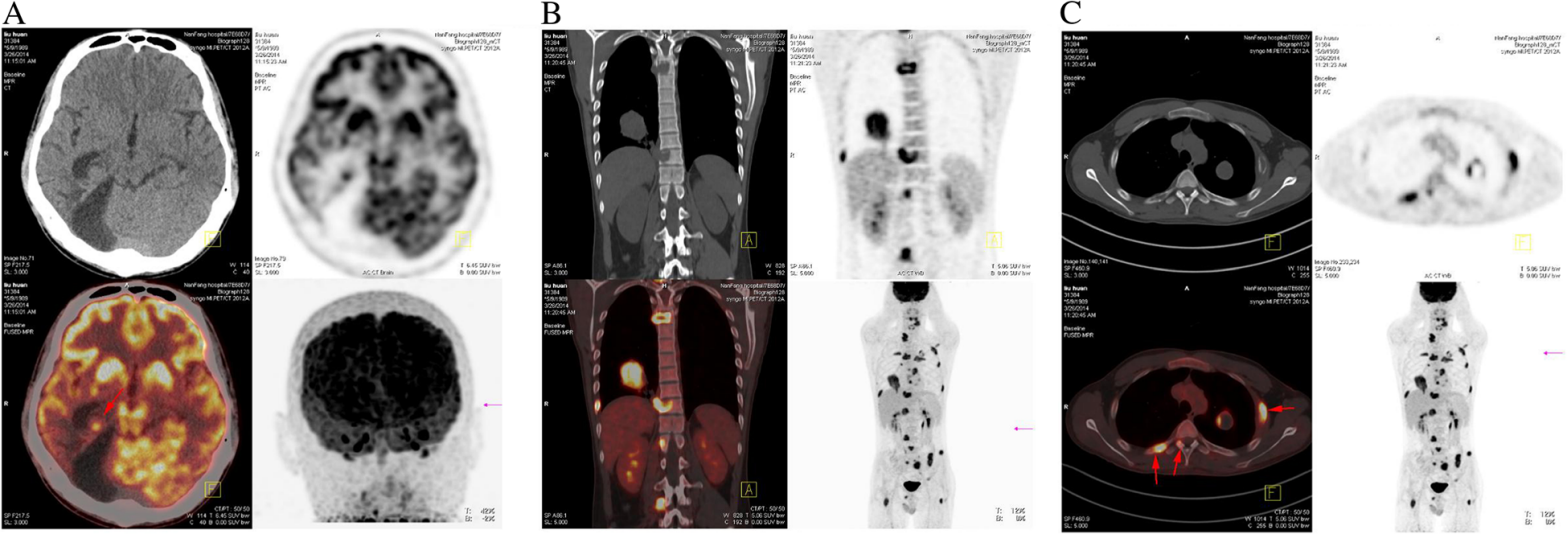
PET/CT findings upon admission. (**a**) Tumor recurrence of the right occipital lobe. (**b**) Hyper-metabolic foci at the lower right lung and the vertebrae. (**c**) Hyper- metabolic foci at the vertebrae and ribs

**Fig. 2:**
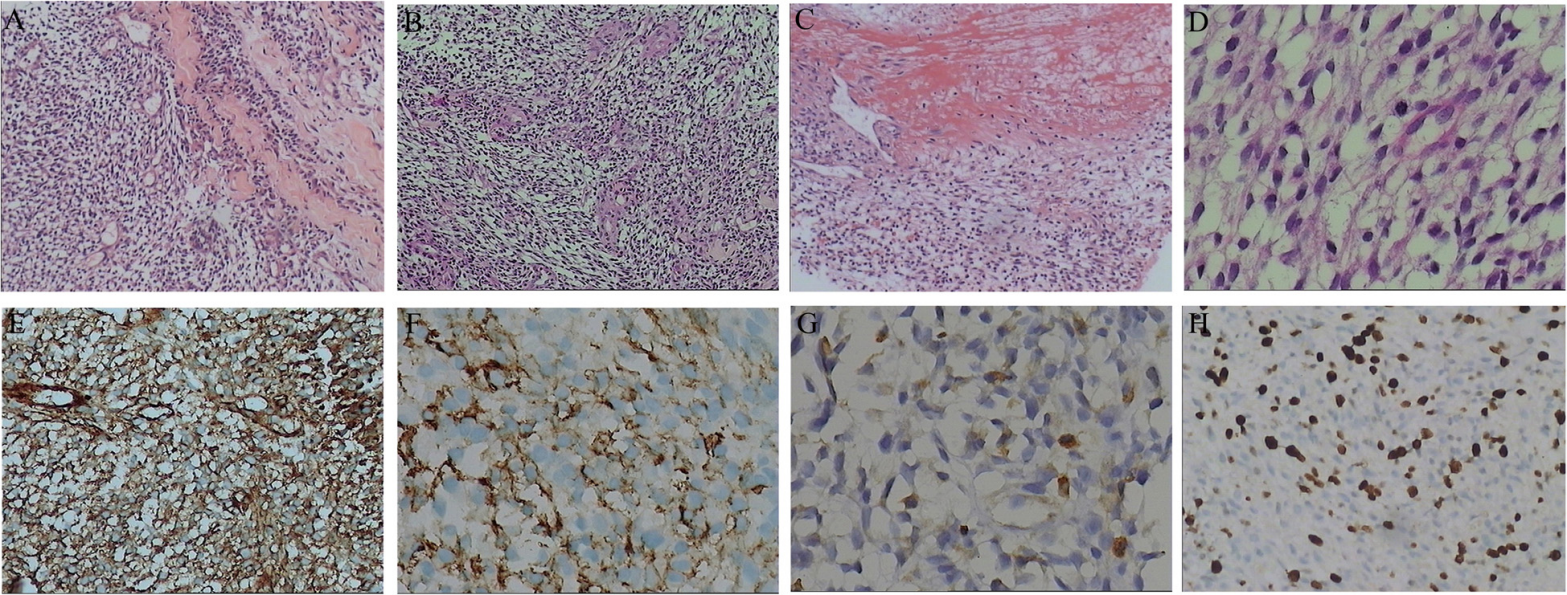
Photomicrographs of the tumor of left-side back of the neck and the third lumbar vertebra. (**a**) The patternless architecture with intervening irregular hyalinzed collagen bundles (H&E, ×100). (**b**) Hypercellularity or hypocellularity and perivascular hyalinization can be seen (H&E, ×100). (**c**) Coagulative necrosis and hemangiopericytoma-like pattern were shown (H&E, ×100). (**d**) Cellular atypia and atypical mitosis were shown (H&E, ×400). (**e**) Tumor cells were diffusely positive for CD99 (immunostain, ×200). (**f**) Tumor cells were focal positive for CD34 (immunostain, ×400). (**g**) Tumor cells were focal positive for BCL-2 (immunostain, ×400). (**h**) Most tumor cells showed Ki-67 labeling index of 40 % (immunostain, ×200)

A 25-year-old male was referred to our hospital with progressive right chest pain and neck pain on March 2014, 4 months after the third time of right-side occipital craniotomy. Initially, he presented with headache, nausea and visual disturbances without obvious cause on December 2012. Magnetic resonance imaging (MRI) revealed intracranial tumor apoplexy of right occipital lobe at local hospital. First right-side occipital craniotomy was performed on 22th December 2012. Postoperation histopathology revealed spindle cell tumor, anaplastic meningioma was a preferred diagnosis. Then he complained of repeat headaches. Follow-up MRI showed a recurrence of tumor in the same location. The second and third open surgery was performed successively on 21th November 2013 and 31th December 2013. Postoperative conformal radiotherapy was administered for two times at doses of 54Gy and 46Gy on13th March 2013 and 17th January 2014, respectively. All these treatments were performed at local hospital. After he was admitted to our hospital, positron emission tomography/computed tomography (PET/CT) showed tumor recurrence of the right occipital lobe (Fig. 1a), several massive or nodular shadows were seen in both lungs, the biggest lesion size was 5.0 cm × 5.1 cm × 5.5 cm (Fig. 1b). The average SUV values were 4.0 in PET scan, with the maximum SUV values was 7.3. Many nodular and patchy thick radioactive lesions were seen in the left attachments of the 5th cervical and the 7th thoracic vertebrae (Fig. 1c), the right attachments of the 1st, 2nd and 5th thoracic vertebrae (Fig. 1b and c), the left 3rd and 6th lateral ribs, the left 6th rear rib, the right 5th and 9th rear ribs (Fig.1c), the right attachments of the 1st and 4th lumbar vertebrae (Fig. 1b), the 5th lumbar vertebral body, the left iliac wing, the sacrum, the right ischium and the upper parts of both femurs. The average SUV value was 4.9, with the maximum SUV value was 8.1. Bone destructions were seen in these lesions under CT scan. Ultrasound guided puncture biopsy of left-side back of the neck and CT guided puncture biopsy of the third lumbar vertebra were performed. Biopsy samples of left-side back of the neck and the third lumbar vertebra showed grayish white or grayish red with irregular shapes. Microscopic findings showed the tumor was composed of atypical cells arranged as fascicular, storiform or patternless pattern with intervening irregular hyalinzed collagen bundles (Fig. 2a). Hypercellularity or hypocellularity, perivascular hyalinization and hemangiopericytoma-like pattern were seen in some areas (Fig. 2b and Fig. 2c). Coagulative necrosis and increased mitotic activity (>4 mitoses/10HPF) were noted in the tumor (Fig. 2c and Fig. 2d). Immunohistochemically, tumor cells were diffusely positive for Vimentin and CD99 (Fig. 2e), focal positive for CD34 (Fig. 2f), Bcl-2 (Fig. 2g) and Actin, negative for CK, EMA, Desmin, CD117, GFAP, PR and S-100. Ki-67 index was more than 40 % (Fig. 2h), consistent with MSFT. Pathologic examination was performed on the HE slides of the right occipital lobe tumor made by former local hospital. Similar morphological change and the same immunohistochemistry phenotype were found. With these features, final pathological diagnosis is MSFT of the right occipital lobe and metastatic MSFT of left-side back of the neck and the third lumbar vertebra.

## Discussion

Although SFT occurs mainly in the mediastinum and visceral pleura, in accordance with previous studies, approximately 30 % of SFT arise in any extrathoracic locations, including the CNS [15]. Since the first SFT case was reported in 1931 by Klemperer and Rabin, it was not until 1996 that the first CNS SFT, located at the meninges, was identified as a distinct entity by Carneiro et al. [16]. The exact origination of CNS SFT remained under controversy. In the CNS, SFTs are usually dura-based, meningioma-like masses and are known to arise from the ubiquitous CD34-positive dendritic interstitial cells of meningeal coverings without meningothelial differentiation [17]. The new World Health Organization classification of tumors of the CNS includes SFT among the mesenchymal nonmeningothelial tumors. It is of interest that most intracranial SFTs seem to be dura-based, whereas two-thirds of spinal SFTs lack a dural attachment [2]. Currently it is more inclined to consider they are derived from vascular mesenchymal cells [17, 18]. Due to the rarity of mesenchymal interstitial tissue in CNS, SFT is a rare tumor of CNS.

SFT in the CNS most occurred in the posterior fossa and spine as well as along spinal nerve rootlets [2]. However, SFT occurrence in brain parenchymal and intraventricular locations has also been rarely described. CNS SFTs occur across all ages with a peak incidence in the fifth and sixth decades of life with near equal gender distribution. A few cases have been described in children. In the current case, the patient was a 23-year old man at the onset of disease. Clinical symptoms and signs of SFT at presentation depend on the location of the tumor and the involved organs or tissues. In Fargen’s review, most SFT in the CNS presented with headache (50 %) followed by gait imbalance (22 %), weakness (19 %), visual loss (19 %), cranial nerve dysfunction (17 %), nausea and vomiting (13 %) and altered mental status or confusion (12 %). It is noteworthy that the current patient presented with rare intracranial hemorrhage (ICH) and similar presentation only was described in 6 literatures [3]. In addition, an association of approximately 4-5 % of pleural SFTs with Doege–Potter syndrome, a rare paraneoplastic syndrome resulting in hypoglycemia from secretion of insulin-like growth factors 2 was not reported in any CNS SFT cases [3, 15].

MSFT showed rapid local recurrence and distant metastasis. Based on the new World Health Organization (WHO) classification of tumors of the CNS published in 2007 and WHO classification of tumors of soft tissue and bone published in 2013, necessary criterions suggestive of the malignant potential of SFTs include hypercellularity, more than 4 mitoses/10 HPFs, variable cytological atypia, tumor necrosis, and/or infilitrative margins, among which mitoses seem to be most prognostic [2, 19]. Some pathologists have suggested that the size of a SFT (bigger than 10cm) is one of the best indicators of malignancy [20]. Currently, there are no criteria for determining malignancy with tumor size in cerebral SFTs. In addition, approximately 8 % of the cases demonstrated “atypical features” based upon brain or bone invasion, marked pleomorphism or high mitotic indices [3]. However, Fargen et al. think it is important to note that above-mentioned atypical features in no way suggest an intermediate classification of SFT between benign and malignant and may merely reflect an increased malignant potential [3]. Even the tumor with benign histopathologic change and intact capsule may develop recurrence, malignant transformation and delayed seeding of the CSF space. While our case showed important malignant evidence such as: obviously nuclear atypia, numerous mitoses, necrosis and infiltrative margins. These histological features were consistent with MSFT.

Most intracranial SFT seem to demonstrate a dural attachment and are usually mistaken for meningioma either radiologically and surgically. Intra-parenchymal non-dural-based SFTs are extremely rare in cranial compartment. As in the current case, first postoperation histopathology revealed a spindle cell tumor, tend to be anaplastic meningioma. Therefore, it is possible that the real frequency of CNS MSFT has been underestimated.

Clinical and imaging features of CNS SFTs are relatively nonspecific and resemble to both meningiomas and hemangiopericytomas, which makes preoperative diagnosis difficult. Definite diagnosis mainly depends on careful examination of histological and immunohistochemical features. WHO classification for tumors of soft tissue treats hemangiopericytoma and SFT as a continuous spectrum of neoplasia, and many lesions that were called hemangiopericytomas prior to 1990 could now be called SFT [3, 15, 19]. Morphologically, SFT is generally characterized by alternating hypercellularity and hypocellularity with spindle cells intervening with irregular hyalinzed collagen bundles showing a pattern-less architecture and a focal hemangiopericytoma-like perivascular pattern riched in staghorn vasculature. However, our case showed mainly hypocellularity due to the nature of puncture tissue. Immunohistochemically, SFT are diffusely positive for CD34, while MSFT are weakened, patchy positive for CD34, which make it difficult to tell MSFT apart from meningiomas. CD34 is not specific for SFTs, as weak and usually patchy staining may be visualized in meningiomas, neurofibromas, and hemangiopericytomas. At this moment, combination of CD34 with CD99 and BCL-2 is necessary for differential diagnosis. Positivity for CD99 and bcl-2 is found in more than half of all cases of SFTs, and strongly stained with the Vimentin is also common. SFTs are usually negative for the S-100, GFAP, EMA, cytokeratin, or vascular antigens. Recent researches validate the diagnostic utility of a novel marker, nuclear expression of STAT6, in distinguishment of SFT from histologic mimics [15]. Moreover, the identification of the NAB2-STAT6 fusion gene can provide important diagnostic information, even in formalin-fixed and paraffin-embedded tissue or when biopsy material is limited [21]. In addition, compared with CD34, ALDH1 expression had a specificity and positive predictive value of 100 % for the diagnosis of SFT [22, 23].

Surgery is the preferred treatment and achieves excellent local control for SFT, however, complete resection of intracranial MSFT is difficult. The prognosis is most likely dependent upon the extent of complete resection rather than histologic grading [2, 3, 15]. Recurrence occurred commonly in cases involving incomplete excision. It is clear that gross total resection (GTR) is superior to subtotal/partial resection (STR) in treatment of CNS SFT, with a nearly 16-fold increased odds of recurrence seen in patients undergoing only STR [3]. Hence, careful follow-up is needed, especially when GTR cannot be achieved. Some scholars suggested intraoperative pathologic examination of CNS SFTs to exclude the residual of tumor at incisal margin. In these cases, adjuvant chemotherapy or radiotherapy may play a role in prevention of recurrence. However, no studies have demonstrated the role of radiotherapy in improving long-term prognosis till now and the number of patients who underwent adjuvant chemotherapy is too small to evaluate any possible benefit [2]. A recent study suggests that Gamma Knife radiosurgery (GKRS) is a feasible adjunct for treating SFT [24]. The current patient exhibited malignant features regarding impossibility of complete resection, rapid recurrence, distant metastasis and histopathological changes. Unfortunately, postoperative radiotherapy is of no effect to him. Adjuvant chemotherapy had been administered to the patient after accurate diagnosis was made and treatment continues till now.

## Conclusion

In conclusion,the authors report here a rare case of MSFT of the right occipital lobe with a rapid recurrence and bilateral lungs, multible bone metastasis. Although intracranial MSFT is extremely rare, it should be considered in the differential diagnosis of tumors in CNS, and carful follow-up is needed. Definite diagnosis depended mainly on pathological morphology and immunohistochemistry.

## Consent

Written informed consent was obtained from the patient for publication of this Case Report and any accompanying images. A copy of the written consent is available for review by the Editor-in-Chief of this journal.
